# Study on the air leakage characteristics of a goaf in a shallow coal seam and spontaneous combustion prevention and control strategies for residual coal

**DOI:** 10.1371/journal.pone.0269822

**Published:** 2022-06-24

**Authors:** Jianwei Li, Xintian Li, Changyou Liu, Ningbo Zhang

**Affiliations:** 1 College of Mining and Coal, Inner Mongolia University of Science and Technology, Baotou, China; 2 School of Mines, China University of Mining & Technology, Xuzhou, China; NUST: National University of Sciences and Technology, PAKISTAN

## Abstract

Air leakage from surface mining-induced fissures can easily cause spontaneous combustion of residual coal in the goaf, which threatens the safe production of the underground working face. In order to study the air leakage law of the goaf under the surface air leakage and the prevention and control technology of spontaneous combustion of residual coal. Based on engineering data from the 6104 working face of the Chuancao Gedan coal mine, this study uses a combination of theoretical analyses, numerical simulations, and field observations to study the dynamic distribution characteristics of the air leakage velocity of surface mining-induced fissures in shallow coal seams, the distribution characteristics of relative pressure, the air leakage velocity, the air leakage flow field, the distribution ranges for the “three zones” of spontaneous combustion in the goaf, and a reasonable range for the pressurized ventilation of the working face. The results show that there is a quadratic relationship between the air leakage speed from the surface mining-induced fissures in shallow coal seams and the distance from the working face. The air leakage speed decreases as the distance from the working face increases, and the air leakage speed in the middle of the working face is slower than the air leakage on either side of the goaf. The pressure difference between the goaf and the surface mining-induced fissures is the root cause of air leakage into the goaf, and a change in the pressure difference has a significant impact on the air leakage flow field and the distributions of the "three zones" of spontaneous combustion in the goaf. When the pressure difference between the ground surface and the working face is maintained within the range of 200~-200 Pa, air leakage is effectively reduced, and the spontaneous combustion of residual coal is inhibited. The research results reveal the air leakage mechanism in the goaf of shallow coal seams and provide a reference for the prevention and control of spontaneous combustion of residual coal in the goaf.

## 1. Introduction

The spontaneous combustion of residual coal in goaf is one of the main disasters threatening the safety of coal mines and a worldwide problem [1, 2]. As is a big country of coal production, China has a serious problem of coal spontaneous combustion in goaf [3]. With decreasing recoverable coal resources in the eastern mining areas of China, the focus of coal resource supply in China has gradually shifted to the western region [4, 5]. The coal seams mined in the western mining areas are generally shallow and most of them are spontaneous and easy to spontaneous combustion coal seams [6, 7]. During the advancement of the working face, a large number of mining cracks are generated in the overlying strata on the working face [8]. Some fissures connect the surface and the goaf and become the air leakage channel between the goaf and the surface [9]. Under the negative pressure ventilation conditions of the mine, the surface air flow continuously supplies oxygen to the goaf, resulting in an increased risk for spontaneous combustion of the residual coal in the goaf [10–12]. At the same time, CO and other toxic and harmful gases produced by the oxidation of residual coal in the goaf flow out from the return airway of the working face under the carry of air leakage, easily causing the CO concentration at the upper corner to exceed the safety limit, posing a threat to safe production [13, 14].

In recent years, scholars have carried out relevant research on air leakage and spontaneous combustion of residual coal in shallow coal seam goaf. Li Ma et al. [15]. studied the distribution characteristics of oxygen concentration in goaf of working face under air leakage in adjacent goaf by field measurement and numerical simulation, and determined the risk zone range of residual coal spontaneous combustion in adjacent goaf. Magdalena Tutak and Brodny [13] simulated the spontaneous combustion environment of residual coal in the goaf by using CFD technology, and analyzed the influence of air leakage intensity and oxygen concentration in the goaf on the spontaneous combustion of residual coal. J. Brodny and M Tutak [2] used Fluent numerical simulation software to divide the risk zone of coal spontaneous combustion in the goaf, and determined the critical value of air leakage flow rate and oxygen concentration leading to the oxidation of residual coal. Zhu et al. [16] analyzed the coupling relationship between coal spontaneous combustion and gas explosion under different air volume of working face by experiments and numerical simulations and determined the influence of air leakage on the explosion danger zone of goaf. Gao et al. [17] used the simulation platform of coal spontaneous combustion in goaf to study the influence of different wind speed on coal spontaneous combustion in working face. Gao et al. [18] studied the spontaneous combustion of coal in the composite goaf formed by shallow and close coal seam mining, and formulated fire-fighting and extinguishing schemes in composite goaf.

Up to now, most researches have focused on the problem of the air leakage and spontaneous combustion of residual coal in goaf, without considering the influence of dynamic change of surface air leakage on spontaneous combustion of residual coal. During the actual advancement of the working face, surface mining fractures present dynamic changes, and their gas conductivity also presents dynamic changes. Therefore, further research is needed on the distribution characteristics of the air leakage flow field in a goaf under the influence of dynamic changes in the air leakage characteristics of surface mining fractures, the distribution characteristics of the "three zones" of spontaneous combustion and the prevention and control technologies for the spontaneous combustion of residual coal.

Based on the engineering data for the 6104 working face of the Chuancao Gedan coal mine, using a combination of field measurements, theoretical analyses and numerical simulations, we studied the distribution of the air leakage flow field and the "three zones" of spontaneous combustion in the goaf under dynamic changes to surface air leakage intensity and proposed a spontaneous combustion prevention strategy based on air leakage flow field control in the goaf. The results provide theoretical guidance and they have significance as an engineering reference for the prevention and control of air leakage into goafs and spontaneous combustion of residual coal during the mining of shallow buried coal seams.

## 2. Engineering background

The average thickness of the coal seam in the 6104 working face of the Chuancao Gedan Coal Mine is 12.8 m, the average dip angle is 5°, and the average burial depth is 116.6 m. The coal seam has a tendency to spontaneously combust and is a grade I coal seam. The spontaneous combustion period is 40~60 days.

**Fig 1:**
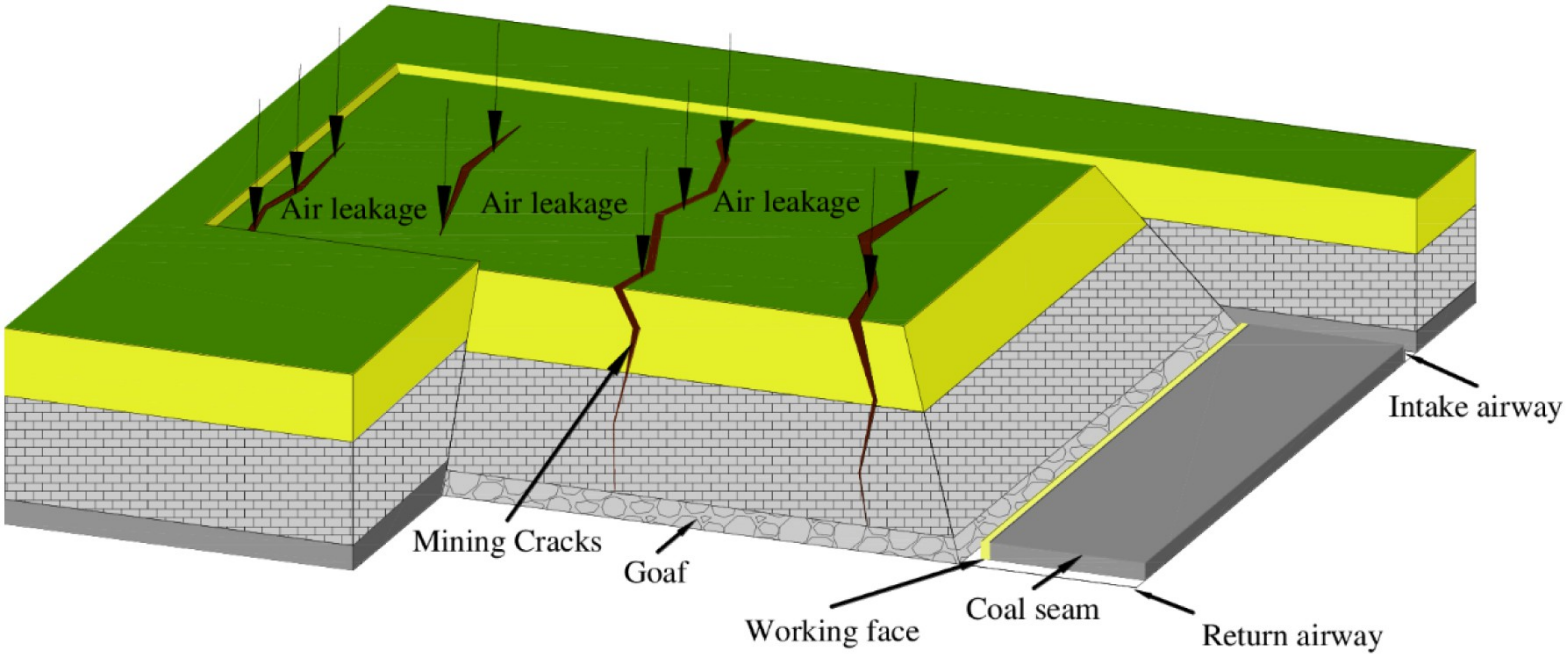
Air leakage from surface mining fractures in the 6104 working face.

The working face of 6104 has a fully mechanized top coal caving mine; the machine mining height is 3.8 m, the coal caving height is 9.0 m, the mining and caving ratio is 1:2.37, the strike length is 2093 m, and the dip length is 148 m. During the advance of the working face, a large number of mining fissures were produced on the surface, and they developed in an arc along the advancing direction of the working face. Due to the effect of negative air pressure, air flows into the goaf through the mining fissures, causing air leakage and ventilation disorders in the working face. The 6104 working face air leakage is shown in Fig 1.

## 3. Air leakage characteristics of the surface mining fractures in shallow buried coal seams

**Fig 2:**
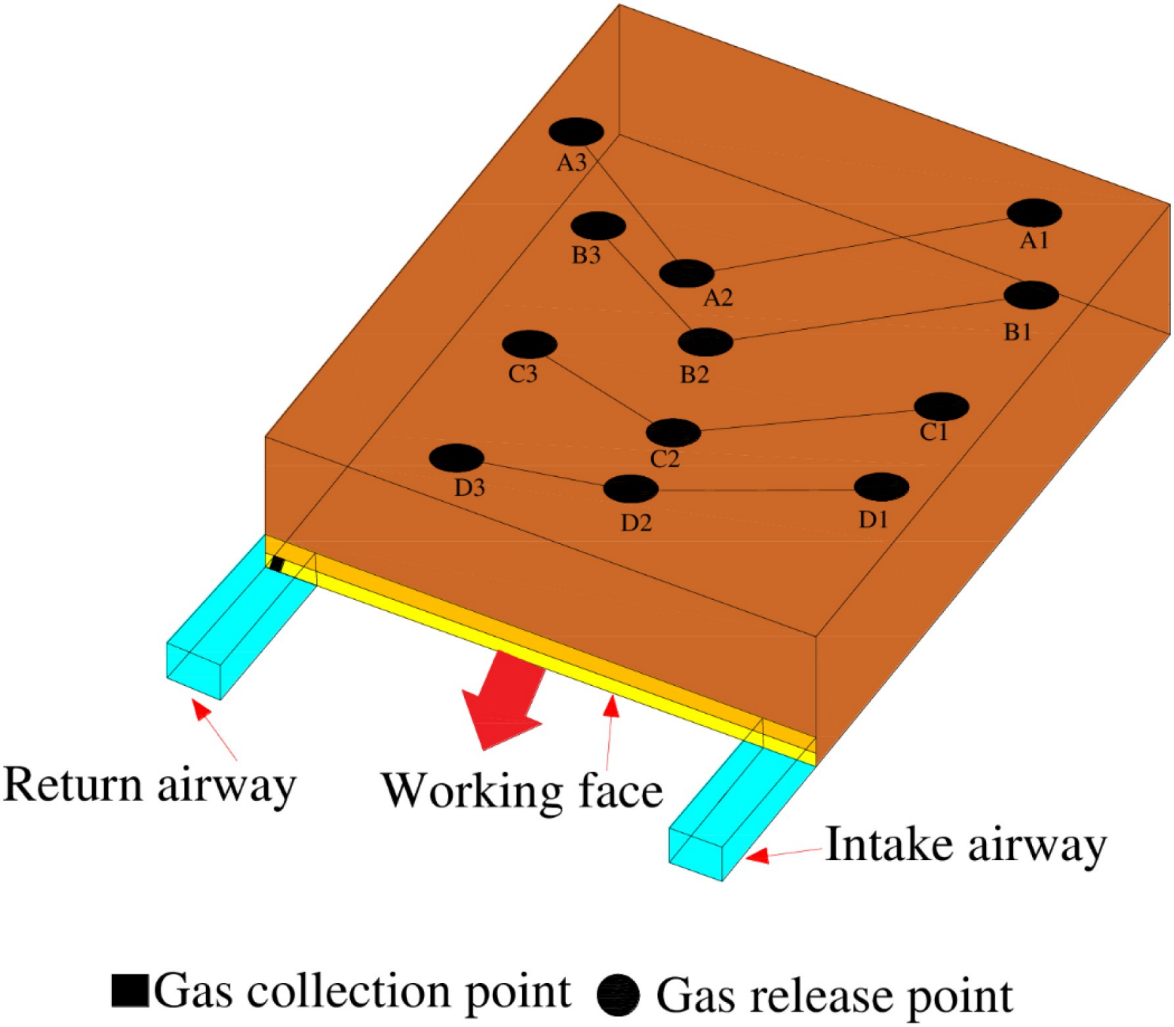
Layout of the SF_6_ release points and collection points.

To study the air leakage characteristics of the surface mining fissures at the 6104 working face, a SF_6_ tracer gas was used for an air leakage test. During this field test, SF_6_ gas was introduced into surface fissures that had large opening volumes and presented a strong possibility for air leakage along the advancing direction of the working face, and release points No. 1~3 were arranged from the inlet side to the return side of the working face. The tracer gas on the return airway side of the working face was detected using a SF_6_ qualitative leak detector, and the time interval between release and detection was recorded. The SF_6_ gas release point arrangement is shown in Fig 2.

Assuming that the air flowed in a straight line from the release point to the return side, the distance *L* between the two points can be calculated from the coordinates of the SF_6_ gas release point and the return detection point.


$$L=\sqrt{{\left(x_{2}-x_{1}\right)}^{2}+{\left(y_{2}-y_{1}\right)}^{2}+{\left(z_{2}-z_{1}\right)}^{2}}$$ (1)

Combining Eq 1 with the time that the SF_6_ gas took to move from the surface release point to the return airway detection point, the air leakage speed can be calculated:

V=L/Δt (2)

In the above equation, *V* is the leakage air flow speed, m/s; Δ*t* is the time interval from release to reception of SF_6_, s. It should be pointed out that due to the bending and staggering of the air leakage channels in the goaf, the actual flow velocity of SF_6_ gas is likely to be greater than the value calculated by Eq (2).

The air leakage test data and the air leakage speed through the surface mining fissures are shown in Table 1.

**Table 1:** Air leakage test analysis.

Release point	Distance from release point to working face/m	Distance from release point to intake roadway/m	Distance from release point to collection point L/m	The elapsed time/s	Air leakage speed/m·s^-1^
A1	-109	26	319	--	0.0000
A2	-61	104	243	1320	0.1843
A3	-82	158	247	2760	0.0894
B1	-83	18	304	3360	0.0905
B2	-53	78	240	780	0.3074
B3	-64	138	231	1260	0.1832
C1	-51	20	299	360	0.8305
C2	-25	65	252	360	0.6991
C3	-21	128	186	240	0.7750
D1	-28	19	280	240	1.1667
D2	-13	73	237	240	0.9880
D3	-5	110	192	180	1.0685

**Fig 3:**
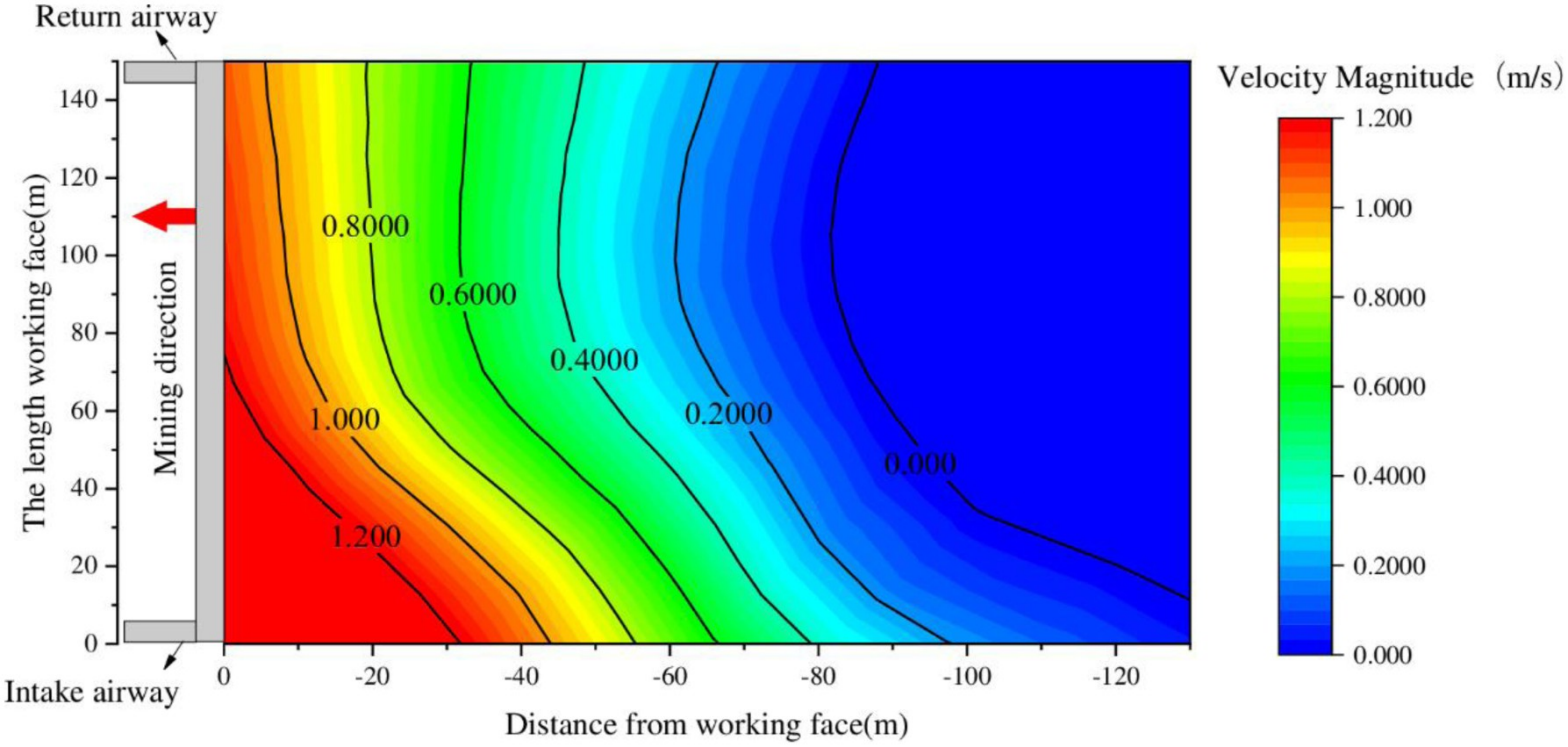
Distribution of the surface air leakage speed contours.

According to the field measured data in Table 1, through the data fitting method, the isoline distribution chart of the air leakage velocity in the surface mining cracks of the 6104 working face is obtained, as shown in Fig 3.

Fig 3 shows that, with the advancement of the working face, the distribution characteristics of the isoline of the air leakage speed of the surface mining fractures were similar to the distribution characteristics of the surface mining fractures. The overall distribution is arc-shaped, and the air leakage speed increased with distance from the working surface. The air leakage speed in the middle of the working face was lower than the air leakage speed in the inlet and return airways, indicating that the cracks in the middle of the working face are more affected by mining than by the fractures on either side of the goaf.

**Fig 4:**
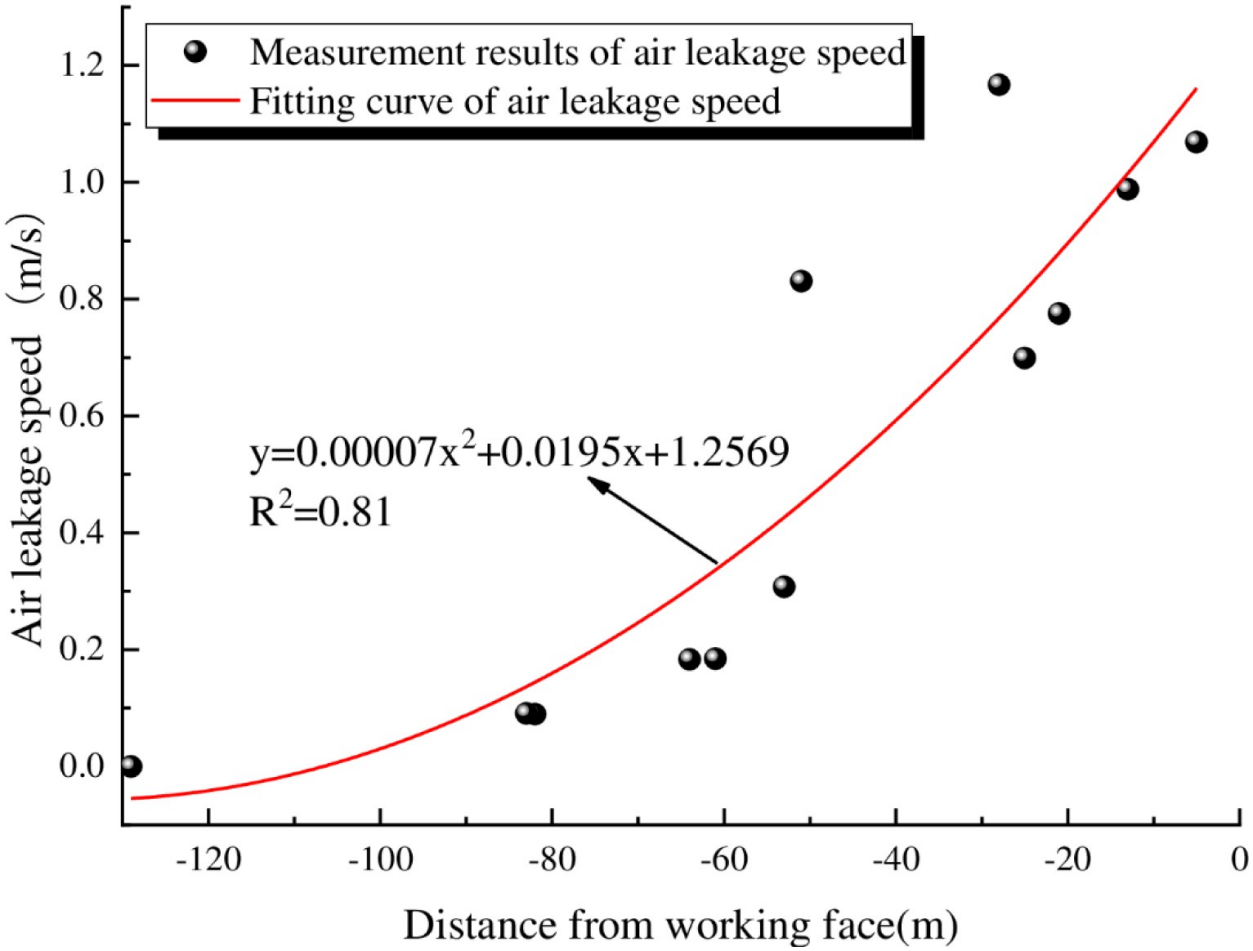
Predicted air leakage speeds at the working face of 6104.

To further analyze the dynamic distribution characteristics of the surface air leakage speed and the distance from the working face, a fitting analysis of the measured data of surface mining fractures and air leakage in the 6104 working face is shown in Fig 4.

Fig 4 shows that there is a quadratic relationship between the air leakage speed of the surface mining fractures and the distance from the working face. With increasing distance from the working face, the air leakage speeds show a decreasing trend. At distances greater than 100 m from the working face, the air leakage speed gradually decreases to 0. The model determination coefficient R^2^ = 0.81, indicating that the goodness of the curve fit is relatively high.


$$y=0.00007x^{2}+0.0195x+1.2569$$ (3)

In the equation, *y* is the fitted surface air leakage speed, m/s; *x* is the distance from the working face, m.

## 4. Numerical simulation of the air leakage flow field in the goaf of a shallow coal seam

### 4.1 Establishment of a numerical calculation model

In this section, based on the actual geological engineering conditions of the 6104 working face, a finite element Fluent numerical simulation software was used to establish a three-dimensional model of the porous medium of the working face of the goaf. Combined with boundary conditions, the pressure, seepage velocity and oxygen distribution in the goaf were obtained by solving discrete algebraic equations, and a three-dimensional numerical simulation of the air leakage flow field in the goaf of the shallow coal seam was carried out.

To facilitate the study of the problem, the following assumptions [19, 20] were made: ①The gas in the goaf is regarded as an ideal incompressible gas; ②The goaf is regarded as an isotropic porous medium; ③The air inlet and return airways are both in the same horizontal plane; the influence of potential energy was not considered. ④The porosity and permeability of the goaf satisfy the spatial position function rather than being a function of mining time.

(1) Establishment of a Mathematical Model

The air leakage in the goaf follows the law of conservation of mass and momentum, and its governing equation is as follows [21, 22]:

$$\frac{\partial (\varphi \rho )}{\partial t}+\frac{\partial (\rho u)}{\partial x}+\frac{\partial (\rho v)}{\partial y}+\frac{\partial (\rho w)}{\partial z}=q\rho$$ (4)

$$\frac{\partial (\rho T)}{\partial t}+\nabla (\rho v)=-\nabla P+\nabla \tau +\rho g-S$$ (5)

In the formula, *φ* is the distribution of pores in the goaf, %; *ρ* is the gas density in the goaf, kg/m^3^; *t* is the time, *s*; *u*, *v*, *w* are gas velocities in the x, y, z directions, m/s; *q* is the intensity of the gas source, s^-1^. *T* is the gas temperature, K; *P* is the gas pressure, Pa; *τ* is the viscous stress tensor; *g* is the acceleration due to gravity, m/s^2^; and *S* is the gas momentum source term.

During the low-temperature oxidation of residual coal in the goaf, the migration of oxygen into the goaf is mainly due to penetration and diffusion. The mass conservation equation is as follows [23]:

$$\frac{\partial C_{O_{2}}}{\partial t}+{\bar{Q}}_{x}\frac{\partial C_{O_{2}}}{\partial x}+{\bar{Q}}_{y}\frac{\partial C_{O_{2}}}{\partial y}+{\bar{Q}}_{z}\frac{\partial C_{O_{2}}}{\partial z}=D_{x}\frac{\partial^{2}C_{O_{2}}}{\partial x^{2}}+D_{y}\frac{\partial^{2}C_{O_{2}}}{\partial y^{2}}+D_{z}\frac{\partial^{2}C_{O_{2}}}{\partial z^{2}}-V_{O_{2}}(T)$$ (6)

In the formula, $C_{O_{2}}$ is the oxygen volume fraction, %; *Q*_*x*_, *Q*_*y*_, and *Q*_*z*_ are the air leakage intensities in the x, y, and z directions, respectively; *D*_*x*_, *D*_*y*_, and *D*_*z*_ are the diffusion coefficients for oxygen migration in the x, y, z directions; and $V_{O_{2}}(T)$ is the coal oxygen consumption when the temperature is T, mol/(m^3^·K).

According to the "O" ring theory, the spatial porosity and the viscous resistance distribution function in the goaf is a function of spatial position. According to Eqs (7) and (8), a UDF for the porous medium’s porosity and permeability in the goaf was compiled [24, 25].


$$n(x,y,z)=\left\{\begin{array}{l} (0.2e^{-0.0223x}+0.1)(e^{-0.15y}+1)\times 1.05^{z},y\leq l/2\\ (0.2e^{-0.0223x}+0.1)(e^{-0.15(l-y)}+1)\times 1.05^{z},y>l/2 \end{array}\right.$$ (7)

$$k(x,y,z)=\frac{D_{p}^{2}}{150}\frac{n{\left(x,y,z\right)}^{3}}{{\left[1-n(x,y,z)\right]}^{2}}$$ (8)

In the formula, *n* is the porosity of any position in the goaf; *k* is the permeability of the goaf, *D*_*p*_ is the average particle size of the rock falling into the goaf, mm; and *l* is the length of the working face, m.

**Fig 5:**
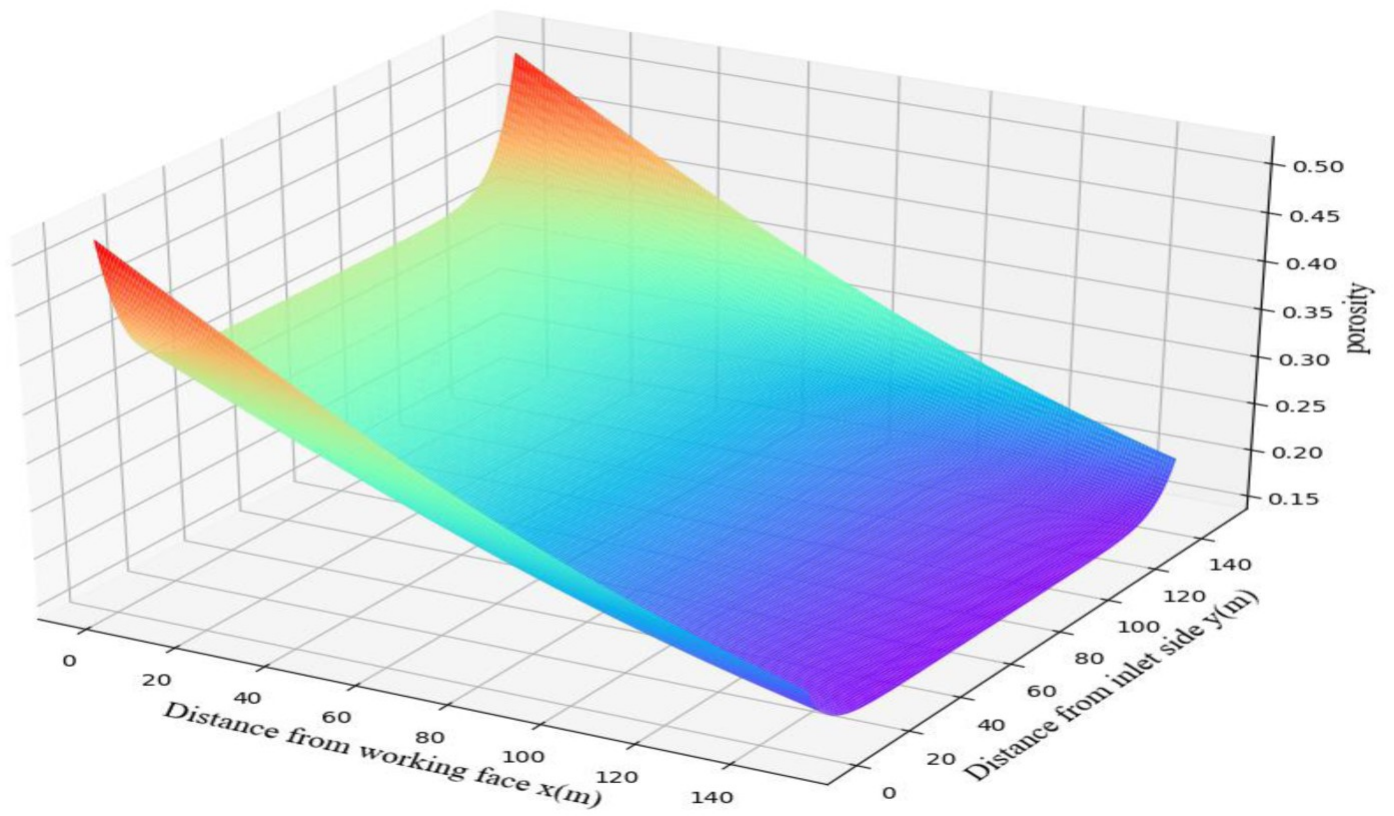
Porosity distribution in goaf.

A mathematical model of the spatial distribution of the goaf porosity was established by Eq (7), and the porosity distribution of the goaf shown in Fig 5 was obtained, which is compiled by a UDF and imported into Fluent for calculations.

(2) Physical Model Construction and Boundary Conditions

Due to the complex distribution characteristics of the surface mining fractures, simulating surface air leakage by constructing equivalent fractures in the physical model cannot truly reflect the dynamic changes in surface mining fracture air leakage as the working face advances. Therefore, by analyzing the air leakage path in the goaf, a suitable location was selected to construct a physical model for dynamic changes in air leakage.

It can be seen from the measurement results for surface air leakage that the direction of air leakage in the goaf is from the surface to the goaf, where the air flow enters the goaf along with the mining fractures, and the mining fractures are only used as the main gas channel for surface air leakage into the goaf. Moreover, the air leakage wind speed measured at the return air side includes the loss of airflow in the mining fractures. Therefore, neglecting the overlying rock cracks in the constructed goaf model will not cause the air leakage characteristics obtained in the previous section to become invalid. At the same time, the collapse zone and the leftover coal in the mined-out area have the characteristics of a porous medium. The air flow through the porous medium is mainly manifested as a continuous movement through the space. A continuous function can be used to realize the dynamic change in the wind speed of the surface air leak. Therefore, we chose to construct a physical model from the caving zone and conducted the simulation of the dynamic change in the air leakage intensity by assuming that the air leakage characteristics of the surface mining fractures remained unchanged.

**Fig 6:**
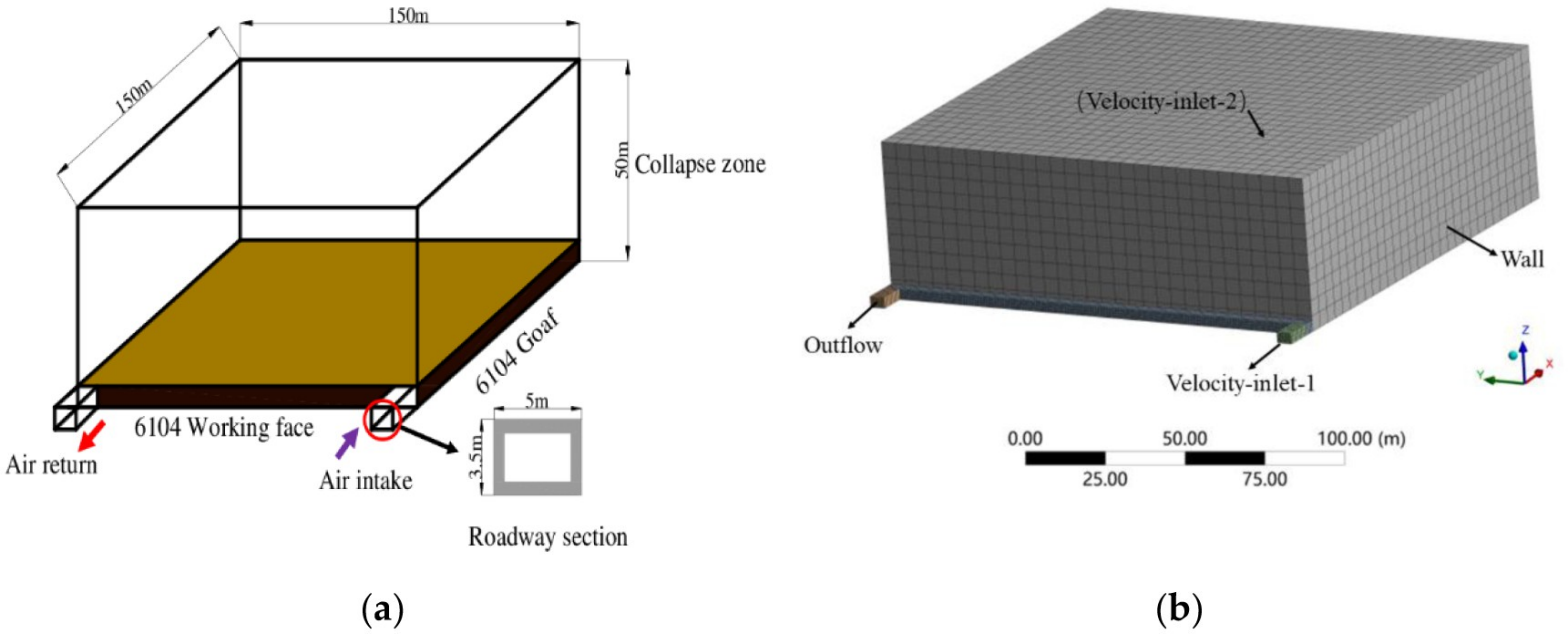
Numerical calculation model of goaf. (a) Physical model face; (b) Meshing.

Based on the above analysis, a three-dimensional numerical model was established, as shown in Fig 6, by simplifying a physical model according to the actual size of the 6104 working face. The width × height of the inlet and return air tunnels were 5 × 3.5 m, the width×height of the working face was 4 × 3.5 m, and the length × width× height of the goaf area was 150 × 150 × 50 m. The whole model was divided by structured hexahedral mesh. Considering that the volume of the goaf was relatively large and the calculation accuracy requirements were low, the size of the calculation unit was divided into 1 m. The working face and intake and return air tunnels were encrypted, and the size of the calculation unit was divided into 0.5 m. The number of meshes divided into the model was 1.15 million, and the mesh quality indexes Maximum cell skewness and Minimum orthogonal quality were 0.2757 and 0.9871, respectively, indicating that the mesh quality was well divided.

In the process of numerical simulation solution, the calculation model should be set first. The numerical model was solved using a pressure-based implicit steady-state solution, and the RNG k-ε model was chosen for the turbulence model. The transport equation of non-chemical reaction components is adopted for the migration of leakage air flow in the goaf. The SIMPLEC algorithm was selected for the model pressure separation solution. The pressure discretization in the control equation adopted the PRESTO format, and the rest adopted the second-order upwind format to improve the convergence accuracy.

Boundary conditions were selected according to the actual situation on site. To realize continuity in the process of the change in the air leakage speed of the mining fractures with the advancement of the working face, a physical model for the collapse zone was established by combining the dynamic changes in air leakage, the quadratic relationship between the air leakage speed obtained from a previous article and the distance from the working face was compiled by UDF. At the same time, to simulate the oxygen consumption environment of the residual coal in the goaf, based on Qin’s [26] closed oxygen consumption experiment of coal samples, it was determined that the oxygen consumption rate of the residual coal was proportional to the ambient oxygen concentration to set the oxygen consumption source item in the goaf.

The upper surface of the model was set as the boundary of the surface air leakage inlet, and the boundary condition was set as the velocity inlet. At the same time, the air inlet roadway was set as the velocity-inlet boundary. According to the 6104 working period, the air distribution rate was 13.6 m^3^/s, and the air inlet velocity was set to 0.78 m/s. The O_2_ mass component of the inlet flow was set at 23% for both surface leakage and inlet velocity. The return roadway was set as the outflow boundary, and the boundary around the area was set as the wall.

When the number of iteration steps is 500 steps, 1000 steps, and 1500 steps respectively, the distribution of the air leakage flow field in the goaf is basically the same. In order to save calculation time, this paper chose to iterate 500 steps. The coefficient y+ was between 30 and 300.

### 4.2 Distribution of the air leakage flow field and spontaneous combustion in the goaf

**Fig 7:**
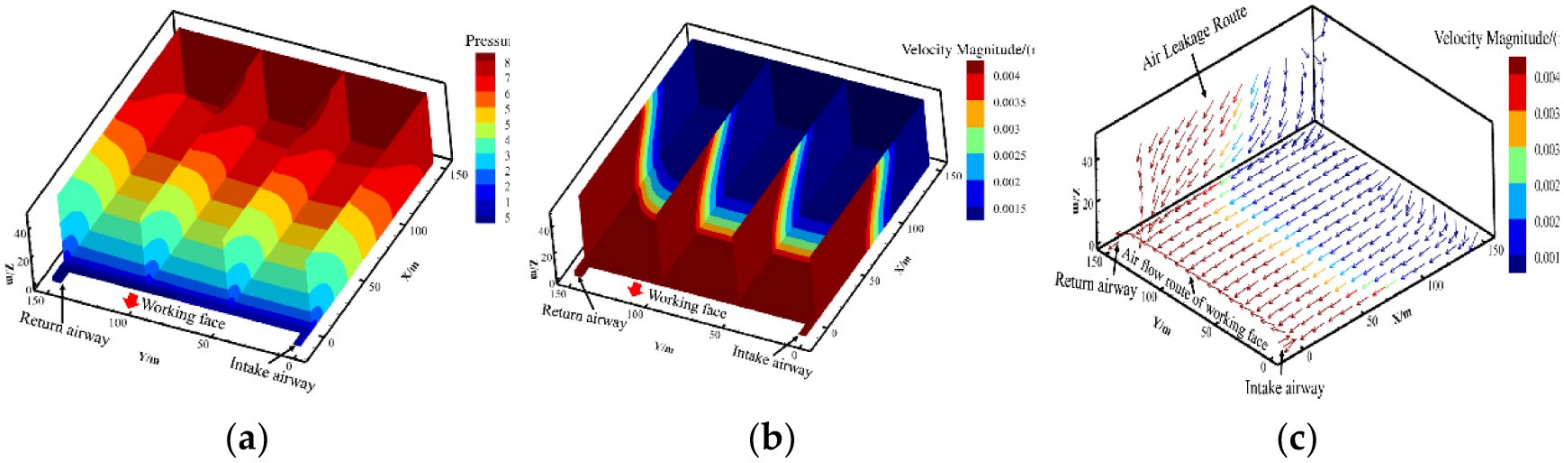
Distribution of the pressure, air leakage velocity and air leakage flow field in goaf. (a) relative pressure; (b) air leakage speed; (c) air leakage flow field.

Under the influence of surface air leakage, the relative pressure, air leakage speed and air leakage flow field distribution in the goaf of the 6104 working face are shown in Fig 7.

Fig 7(a) shows that under the influence of surface air leakage, along the positive z-axis, relative pressure increases with increasing goaf height; along the positive x-axis, relative pressure increases with increasing distance into the goaf. Considering the pressure difference between the surface and the working face, the directions of air leakage were determined to be the surface direction of the goaf and the goaf to the working face.

Fig 7(b) shows that under the influence of surface air leakage, along the positive z-axis, surface air leakage velocity increases with increasing goaf height; along the positive x-axis, surface air flow velocity decreases with increasing distance into the goaf. At the same time, due to the different degrees of compaction between the middle of the working face and the sides of the goaf, there is a certain difference in the air leakage velocity between the inlet and return air side of the goaf and the middle of the working face.

Fig 7(c) shows that under the effect of the pressure difference between the surface and the working face, fresh air flow enters the goaf and the working face through the surface mining fissures, meets the working face air flow, and flows out from the return air channel. From the color profiles of the air leakage trace, the air leakage speed decreases with increasing distance from the working face. The surface air leakage intensity near the working face is greatest, which is the main surface air leakage area. The air leakage speed at the depth of the goaf is 0.

**Fig 8:**
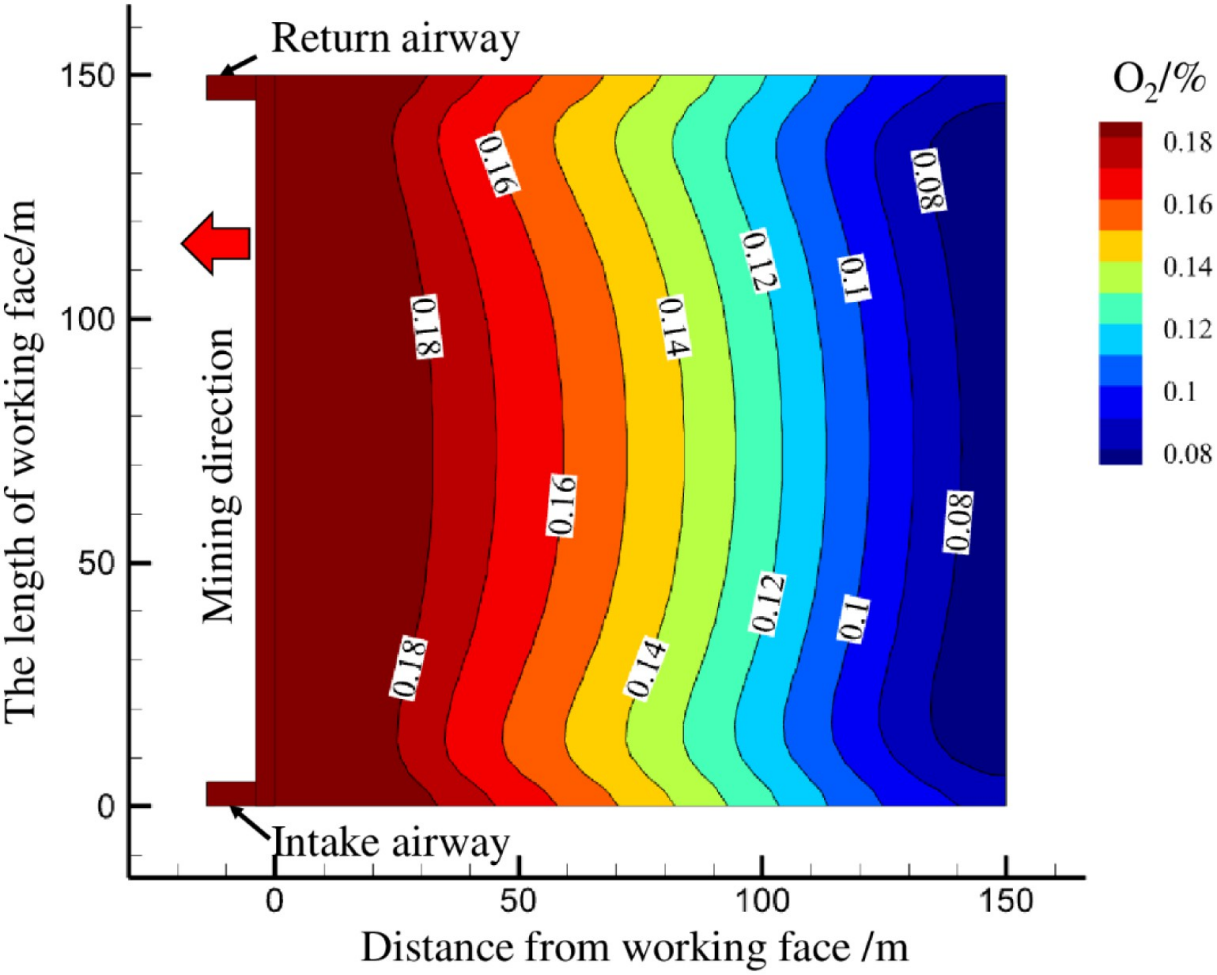
Distribution of oxygen concentration in the goaf.

Fig 8 shows the simulation results of the oxygen concentrations in the goaf. The oxygen concentration method is used to divide the risk area for spontaneous combustible residual coal in the goaf [27]. Fig 8 shows that the range 0~33 m from the working face is the heat dissipation zone with an oxygen concentration greater than 18%. This area is close to the mining area of the working face. The surface air leakage is relatively strong, and fresh air flow enters the goaf through the cracked channel and takes the heat generated by the oxidation reaction of the residual coal out of the goaf, which makes heat accumulate from the oxidation of the coal difficult and lowers the risk for spontaneous combustion of the residual coal. The range 33~140 m from the working face is a zone of increasing oxidation temperature with oxygen concentrations between 8% and 18%. This area has sufficient oxygen supply conditions and a good heat storage environment and is a dangerous area with a high incidence of spontaneous combustion of residual coal. One hundred forty meters beyond the lagging working face is the suffocation zone where the oxygen concentration is below 8%. The oxygen concentration in this area is low and is not enough to maintain the spontaneous combustion and temperature increase for the residual coal, and the oxidation reaction of the residual coal essentially ends.

**Fig 9:**
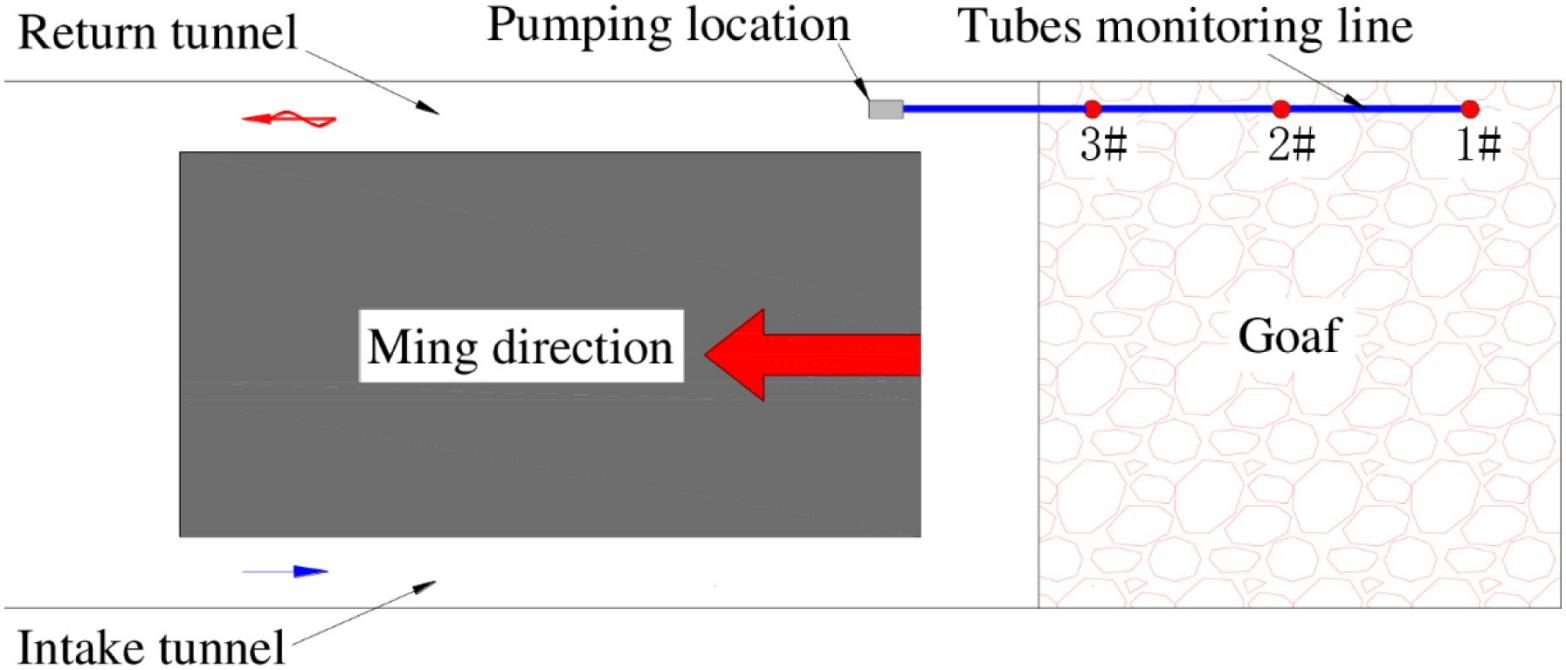
Layout of measuring points of pre-embedded beam tube in working face.

The gas in the goaf is collected by the pre-embedded beam tube extraction method in the goaf, the concentration of each component of the gas in the goaf is analyzed by a gas chromatograph. In the return roadway pre-embedded beam pipes of the working face, a total of 3 measuring points were arranged, the distance between the measuring points was 50m, and they were numbered 1~3#. The 4.0-inch steel tube was used to protect the beam tube from damage. The specific arrangement is shown in Fig 9.

**Fig 10:**
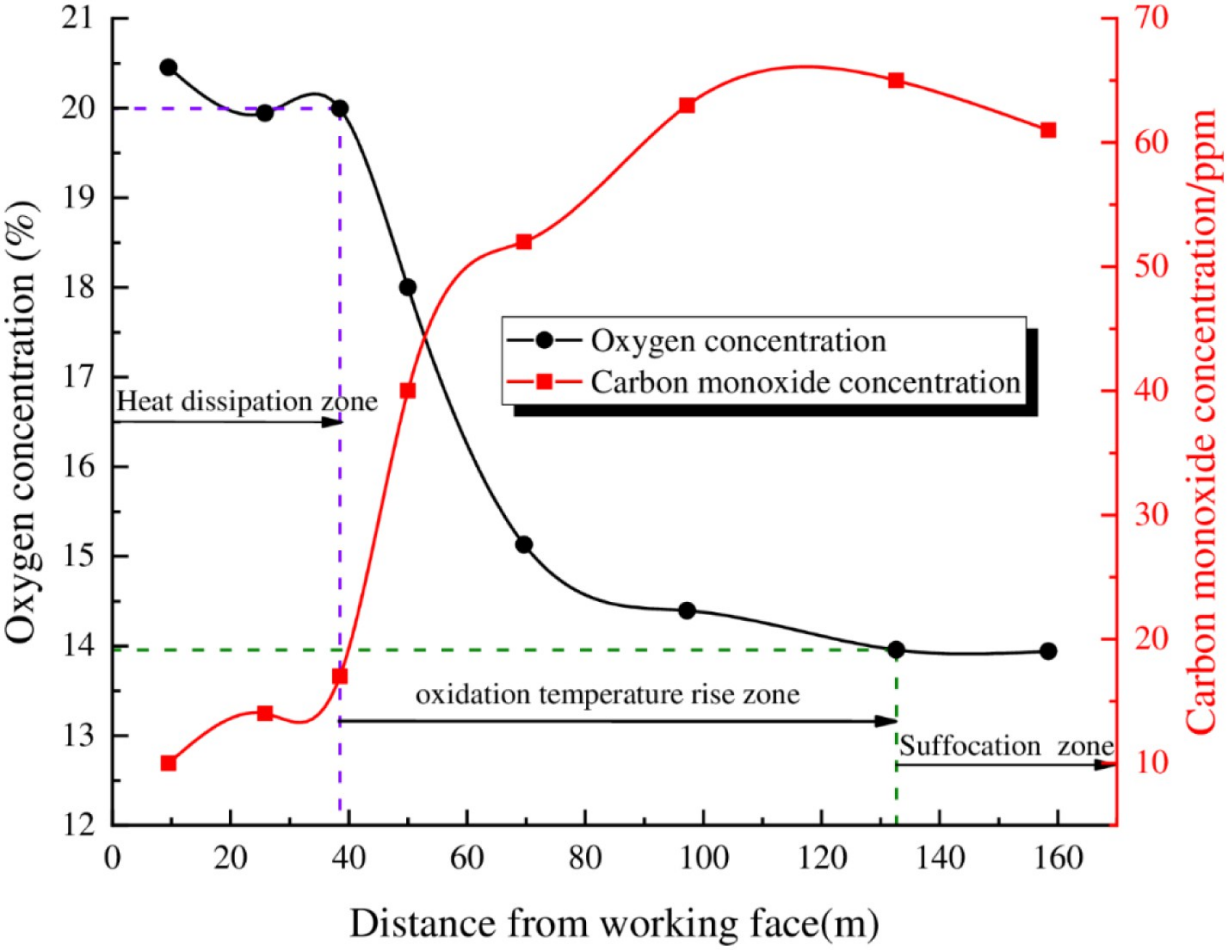
Distribution of O_2_ and CO at different locations in the goaf.

The changes of oxygen and carbon monoxide concentrations in the 6104 goaf with the advancing distance of the working face are shown in S1 Table and Fig 10, respectively.

Fig 10 shows that, as the working face advances, the O_2_ concentration in the goaf continues to decrease, and the CO concentration gradually increases. Within a range of 0~38 m from the working face, the oxygen concentration fluctuates between 20.4%~19.9%, the CO concentration is between 10~17 ppm, and there is no obvious increasing trend. This shows that the area is close to the working face, air leakage is strong, the air leakage will remove the CO produced by the oxidation of the residual coal, and there is no obvious increase in CO concentrations. Within a range of 38~133 m from the goaf, the oxygen concentration in the goaf drops sharply from 19.9% to 13.9%. At the same time, the CO concentration in this range increases sharply, from 17 ppm to 65 ppm. This indicates that the residual coal in this area undergoes a violent oxidation reaction, and the air leakage intensity is reduced, so it is difficult to remove the CO produced by the oxidation of the residual coal, causing the CO concentration to increase. Within 133 m of the lagging working face, the oxygen concentration remains at 13.96%, and the CO gas concentration gradually decreases below 65 ppm, indicating that the oxidation process of the residual coal in this area is essentially over, the generation of CO gas is suppressed, the CO gas gradually disperses, and the CO concentration shows a downward trend. In summary, it is determined that the heat dissipation zone of the goaf is 0~38 m, the oxidation temperature rise zone is 38~133 m, and the suffocation zone is beyond 133 m.

The numerical calculation results are largely consistent with the distribution ranges for the "three zones" of spontaneous combustion in the goaf measured onsite, indicating that the air leakage boundary of the numerical calculation model is controlled by the air leakage function of surface fissures, which fits the actual observations for air leakage in the 6104 working face. The model predicts the impact of the dynamic changes in surface fissures air leakage with the advancement of the working face on the air leakage flow field and the distribution range for the "three zones" of spontaneous combustion in the goaf.

## 5. Pressurized ventilation of the working face to prevent spontaneous combustion of residual coal in the goaf

### 5.1 Numerical simulations of pressurized ventilation in the working face

Negative pressure ventilation is adopted in the 6104 working face, and the pressure difference between the working face and the leakage fissures is the root cause of air leakage into the goaf. Therefore, pressurized ventilation was selected to reduce the pressure difference between the ground surface and the mining face to reduce air leakage into the goaf, suppress the spontaneous combustion of the residual coal in the goaf, and prevent the accumulation of CO and other toxic and harmful gases in the upper end or gushing to the working face, thereby ensuring safe mining in the working face [28–30].

**Fig 11:**
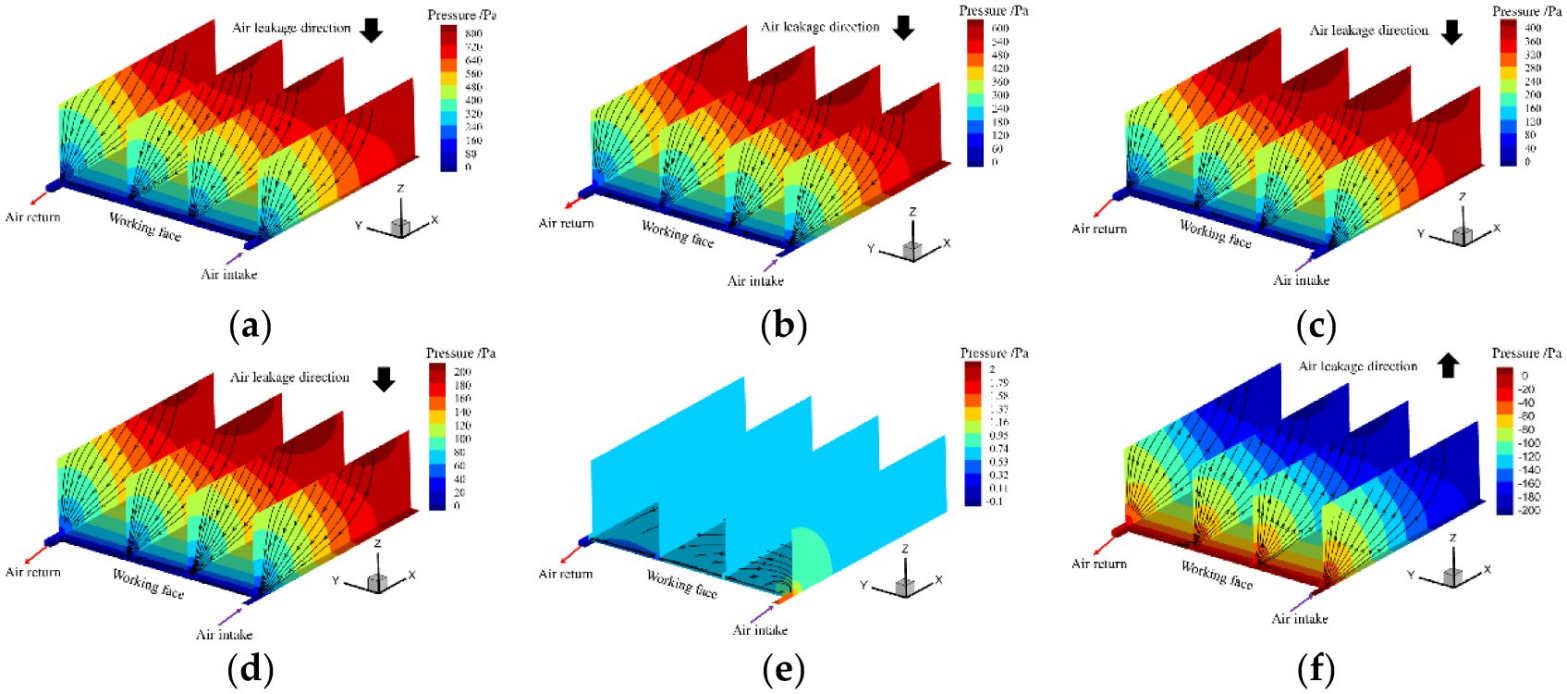
Distribution of pressure and air leakage flow field in goaf under different pressure differences between the ground surface and mining face. (a) ΔP = 800 Pa; (b) ΔP = 600 Pa; (c) ΔP = 400 Pa; (d) ΔP = 200Pa; (e) ΔP = 0 Pa; (f) ΔP = -200 Pa.

When the working face adopts pressurized ventilation measures, if the absolute pressure of the working face is too small, pressurized ventilation measures struggle to achieve the expected effects. If the absolute pressure of the working face is too high, it will become the air supply to the goaf, which is not conducive to the prevention and control of the spontaneous combustion of the residual coal in the goaf. Therefore, it is necessary to determine the proper range of pressurization for ventilation for working face pressurized ventilation. Based on the above analysis, the above numerical calculation model is used to analyze the relative pressure distribution and the air leakage flow field distribution of the pressure difference between the ground surface and the mining face in the goaf from 800 Pa~-200 Pa, as shown in Fig 11.

Fig 11 shows that as the working face is gradually pressurized, the pressure difference between the ground surface and mining face continues to decrease. When the pressure difference between the ground surface and mining face is 800 Pa~0 Pa, the surface air leakage will flow from the surface mining-induced fissures to the goaf under the action of the pressure gradient and finally converge with the air flow of the working face and flow out of the return airway side. When the pressure difference between the ground surface and mining face is approximately 0 Pa, the pressure difference disappears, and the air leakage is basically 0. The air flow in the working face flows in from the air inlet channel and flows out from the return air channel. When the pressure difference between the ground surface and mining face is 0~-200 Pa, the local pressure at the working face is too large, which causes the air at the working face to flow into the goaf and finally flow out from the surface mining-induced fissures.

**Fig 12:**
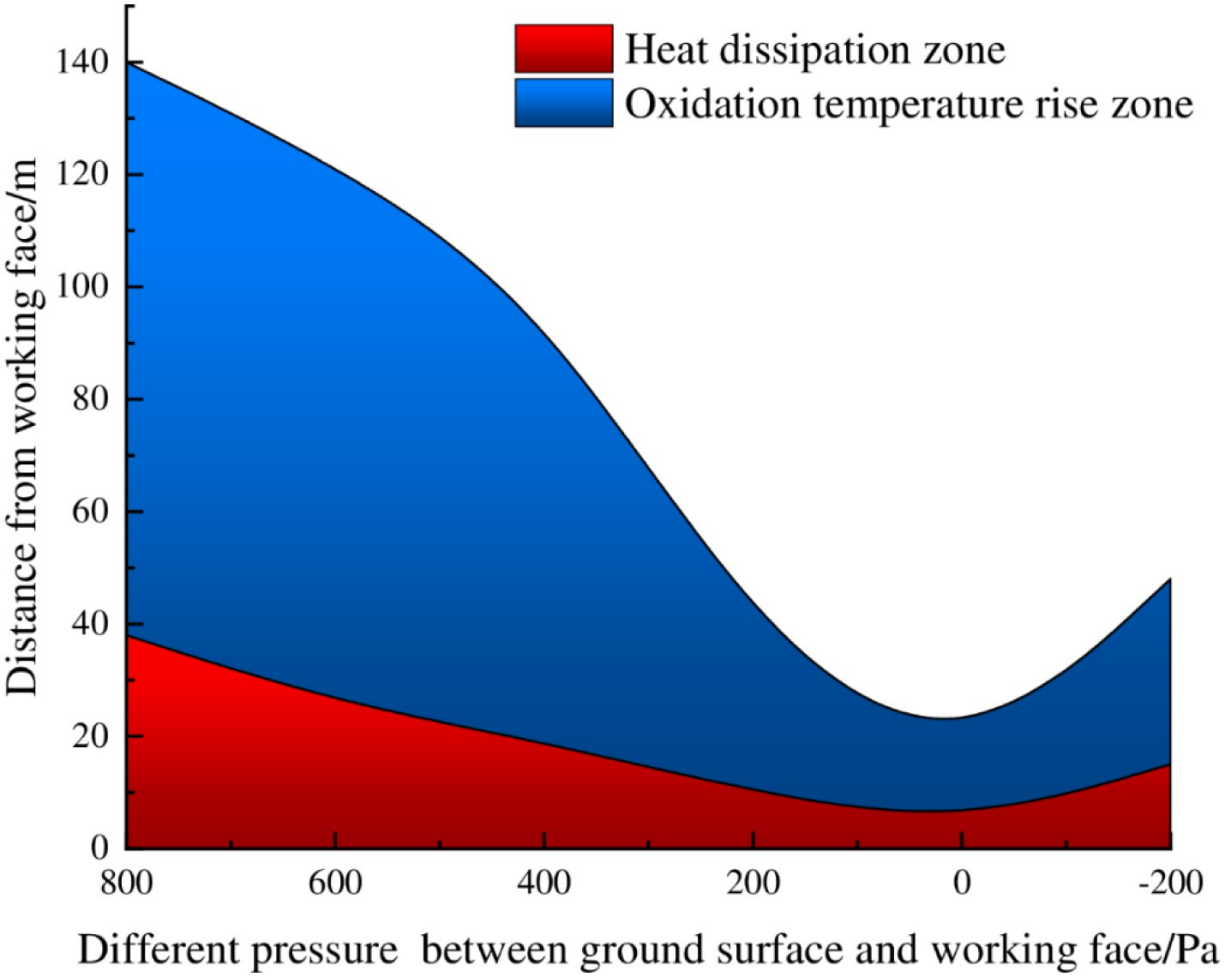
"Three zones" of spontaneous combustion in the goaf under different pressure differences between the mining face and ground surface.

Based on the numerical simulation results for the air leakage velocity and oxygen concentration distribution in the goaf under different pressure differences between the ground surface and the mining face, the dual indicators of air leakage velocity and oxygen concentration are used to divide the risk area for the spontaneous combustion of residual coal in the goaf, and the "three zones" distribution for spontaneous combustion in the goaf is shown in S2 Table and Fig 12, respectively.

Fig 12 shows that after pressurized ventilation is applied to the working face, as the pressure difference between the ground surface and the mining face decreases, the range of the heat dissipation zone and the oxidation temperature rise zone in the goaf changes in stages. When the pressure difference between the ground surface and the mining face is 800 Pa~0 Pa, the range of the heat dissipation zone and the oxidation heating zone decreases with decreasing pressure difference. When the pressure difference is 0~-200 Pa, the range of the heat dissipation zone and the oxidation heating zone increases with decreasing pressure difference. Therefore, according to the distribution range of the “three zones” of spontaneous combustion in the goaf with different pressure differences between the mining face and ground surface, it is determined that, when the pressure difference of the working face is maintained at 200~-200 Pa, the width of the oxidation temperature rise zone remains at a relatively low range, slowing the spontaneous combustion rate of residual coal in the goaf.

### 5.2 Applications in the field

The 6104 working face of the Chuancao Gedan coal mine employs negative pressure ventilation, and the measured air leakage onsite is 3.76~10.5 m^3^/s. With the continuous advancement of the working face, the openings of the surface mining fractures gradually increase, and their length gradually increases. The development of surface mining fractures leads to a continuous increase in air leakage, the CO concentration gradually increases, and the risk of spontaneous combustion of coal increases in the goaf. According to the actual conditions of the site, regulation windscreens, local ventilators and other facilities are combined, and a 6104 working face pressurized ventilation system is proposed as follows:

1. Utilize the existing regulation windscreens of the 6104 working face to achieve controllable pressurization through reinforcement. At the same time, measure and adjust the air volume, pressure, and gas concentrations of the main location in the pressurization ventilation system over time according to test results to ensure the effective operation of the pressurized ventilation system.
2. At the 6104 working face, use a "U"-type supercharged ventilation system with 4 sets of 55 kW fans, 2 sets for use and 2 sets for standby. The fan must operate with an "automatic switching" function to ensure that the main running fans will automatically switch to the standby fans if the main running fans fail. Installed the fans in the connecting channels of the #6 coal main transportation roadway and the main auxiliary transportation roadway, and set the air flow rate to 800 m^3^/min.
3. Start the local ventilator to supply air to the 6104 working face, and reduce the pressure difference between the mining face and the ground surface on the premise that the air volume of the original working face will remain unchanged. According to the measured inlet and return air volume, the pressure adjustment methods for the fans and air windows are used to change the pressure distribution of the 6104 working face to reduce surface air leakage, suppress CO gas emission and prevent the spontaneous combustion of residual coal.

**Fig 13:**
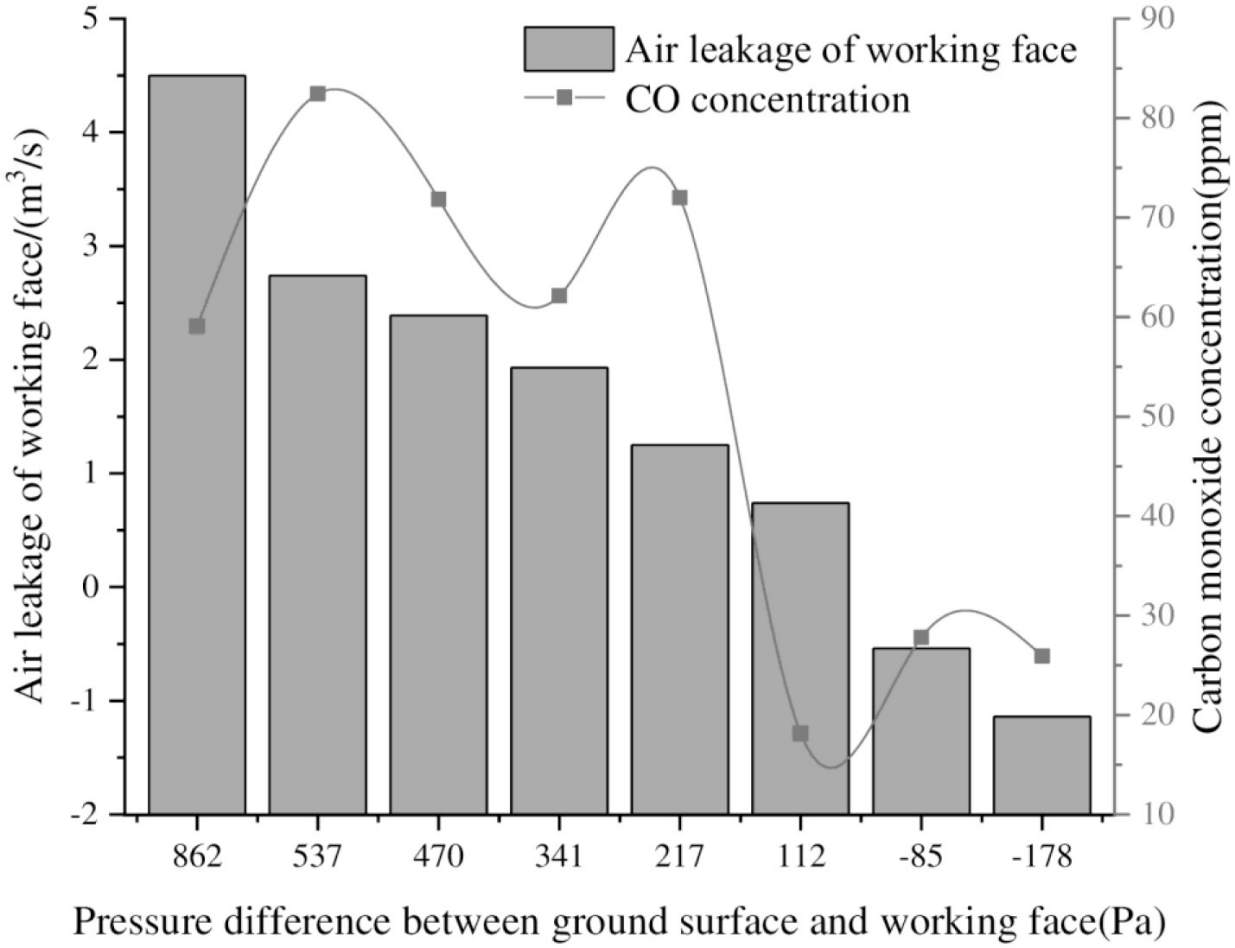
Air leakage of the working face and upper corner CO concentration during pressurization.

To verify the reliability of the simulation results, the model analysis results were verified by measured field data. During the pressurization process for the 6104 working face, a DYM3 empty box barometer and a SF6 quantitative leak detector were used to obtain the pressure difference between the surface and the working face and the air leakage at the working face, respectively. At the same time, an upper end CO monitor was used to simultaneously monitor the changes in CO concentrations at the upper end of the return air and plot the surface air leakage and the changes in the upper end CO concentrations during the pressurized ventilation process, as shown in S3 Table and Fig 13, respectively.

Fig 13 shows that during the pressurization process of the 6104 working face, as the pressure difference between the ground surface and the working face decreases, the air leakage of the working face and the CO concentration at the upper end of the return air gradually decrease. When the pressure difference between the surface and the working face is positive, the working face air leakage and CO concentrations will decrease as the pressure difference decreases. When the pressure difference between the surface and the working face is negative, the air leakage of the working face increases with the increase in pressure difference, and the CO concentration at the upper end is affected by the change in the direction of air leakage, and it still shows a decreasing trend.

**Fig 14:**
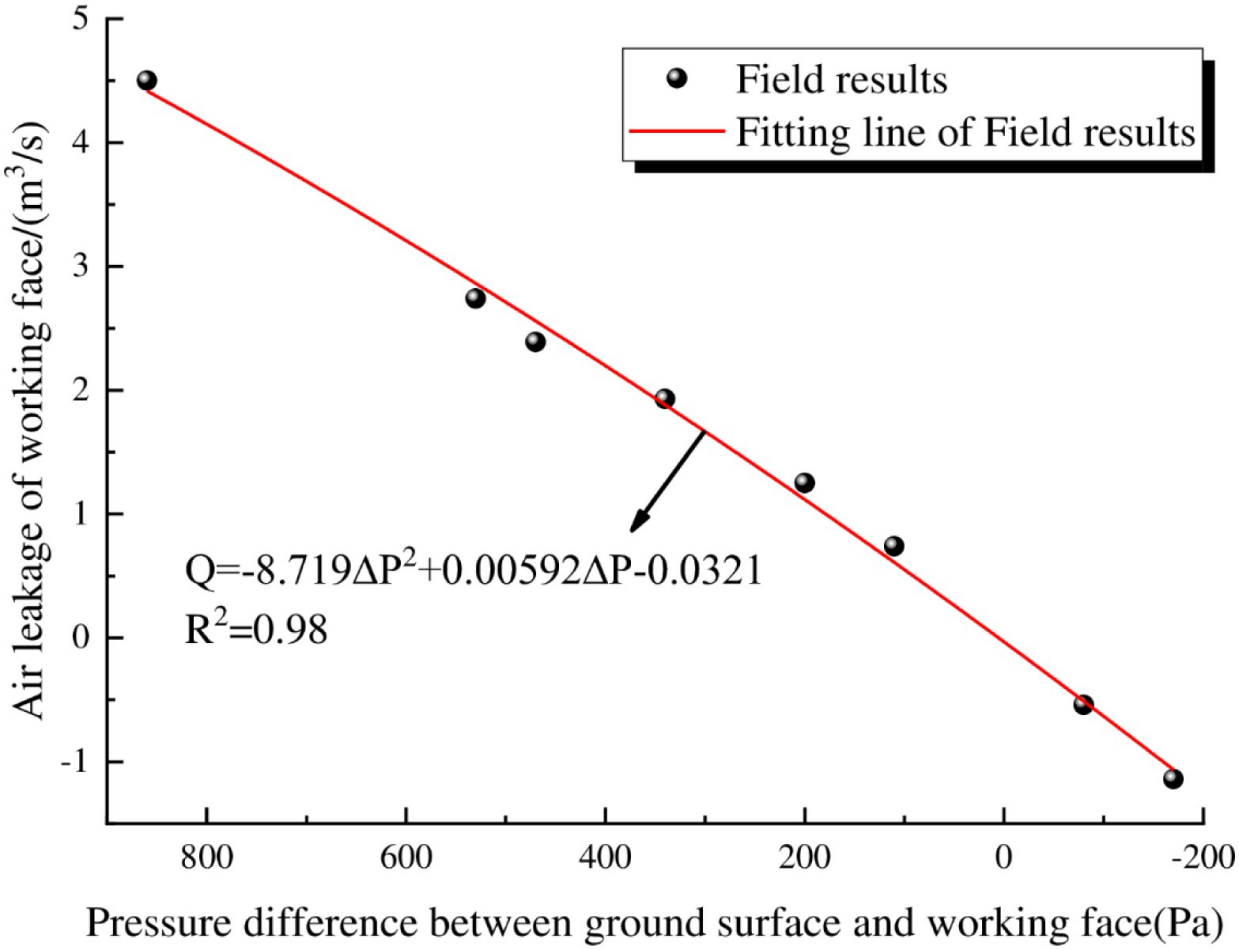
Prediction of air leakage in the 6104 working face.

Fig 14 shows that the air leakage at the 6104 working face decreases with a decrease in the pressure difference between the surface and the working face, indicating that with the effective implementation of the pressurized ventilation system, the pressure difference between the surface mining fractures and the working face approaches 0, and the air leakage of the working face is reduced. Through regression analysis, the correlation function between the pressure difference between the surface mining fracture and air leakage of the working face is obtained:

$$Q=-8.719\Delta P^{2}+0.00592\Delta P-0.0321$$ (9)

In the above equation, *Q* is the air leakage in the goaf of the 6104 working face, m^3^/s; ΔP is the pressure difference between the ground and the working face, Pa. The regression coefficient of the model is 0.98, which indicates that the equation has a high goodness of fit, meets the actual needs of the project, and provides a theoretical basis for adjusting the air leakage at the working face during actual operations.

Based on the field test data and numerical simulation results, through analysis of air leakage at the working face and CO concentrations of the spontaneous combustion products of residual coal, it is determined that when the pressure difference between the surface and the working face is maintained at 200~-200 Pa, the air leakage in the goaf can be effectively reduced, and the spontaneous combustion of residual coal can be suppressed.

## 6. Research limitations and future research avenues

In this paper, the air leakage velocity of different surface cracks is measured by using the tracer gas measurement technology of surface mining cracks and the surface leakage function is obtained by numerical fitting. Besides, the surface air leakage function is compiled by using UDF in Fluent to realize the continuous change of surface air leakage, and simulate the air leakage law and spontaneous combustion "three zones" distribution in the goaf under the dynamic change of surface air leakage.

Some conclusions have been reached during the thesis research, but there are still some limitations. In constructing the geometric model of goaf, the surface leakage process was simplified and a surface leakage mining area model was constructed from the collapse zone. The air leakage flow field in the goaf and the "three zones" distribution of spontaneous combustion in the goaf under the actual occurrence of actual mining fissures should be further studied.

As for the technical measures of preventing and controlling residual coal spontaneous combustion, this paper only studies the technology of preventing residual coal spontaneous combustion in goaf by using working face pressurized ventilation. The next step should be the surface fracture plugging, nitrogen injection and other goaf remaining coal control methods for in-depth study.

## 7. Conclusions

1. There is a quadratic relationship between the air leakage speed at the surface mining-induced fissures in shallow coal seams and the distance from the working face. The air leakage speed decreases with increasing distance from the working face, and the air leakage speed in the middle of the working face is less than the air leakage speed on both sides of the goaf.
2. The combined use of Fluent and UDF to compile a surface air leakage function can accurately realize the inversion simulations for relative pressure, air leakage velocity, and the air leakage flow field in the goaf by assuming a continuous change in the air leakage velocity of the surface mining-induced fissures with the advancement of the working face.
3. The pressure difference between the goaf and the surface mining-induced fissures in shallow coal seams is the root cause of the air leakage in the goaf. The change in the pressure difference between the mining face and the ground surface has a significant impact on the air leakage flow field and the distribution of spontaneous combustion in the goaf.
4. During the pressurization process for the 6104 working face, as the pressure difference between the ground surface and the working face decreases, the air leakage of the working face and the CO concentrations at the upper end of the return air gradually decrease. The working face pressurization is maintained at a pressure difference in the range of 200~-200 Pa between the surface and the working face, which can effectively reduce air leakage at the working face and suppress the spontaneous combustion of residual coal in the goaf.

## Supporting information

S1 Table The oxygen concentration and CO concentration in goaf at different distances from working face are obtained according to the measured results of goaf bundle tube. (DOCX)

S2 Table According to the numerical calculation model, the three-zone spontaneous combustion in the goaf under different pressure differences between the upper and lower wells obtained from the simulation. (DOCX)

S3 Table According to the field measurement results, the air leakage of the working face and the CO concentration in the upper corner during the pressurization process are obtained. (DOCX)
