# Supplementary material for: Study on the air leakage characteristics of a goaf in a shallow coal seam and spontaneous combustion prevention and control strategies for residual coal
Source: PLoS One. 2022 Jun 24;17(6):e0269822. doi: 10.1371/journal.pone.0269822 (PMC9232134; doi:10.1371/journal.pone.0269822)
Supplement: S1 Table — (DOCX) [file pone.0269822.s001.docx]

S1A Table The oxygen concentration and CO concentration in goaf at different distances from working face are obtained according to the measured results of goaf bundle tube

| Distance from working face(m) | Oxygen concentration(%) | Carbon monoxide concentration(ppm) |
| --- | --- | --- |
| 9.5 | 20.4537 | 10 |
| 25.85 | 19.9434 | 14 |
| 38.5 | 19.9942 | 17 |
| 50 | 18 | 40 |
| 69.7 | 15.1293 | 52 |
| 97.25 | 14.3942 | 63 |
| 132.65 | 13.9561 | 65 |
| 158.4 | 13.9414 | 61 |
