# Supplementary material for: Study on the air leakage characteristics of a goaf in a shallow coal seam and spontaneous combustion prevention and control strategies for residual coal
Source: PLoS One. 2022 Jun 24;17(6):e0269822. doi: 10.1371/journal.pone.0269822 (PMC9232134; doi:10.1371/journal.pone.0269822)
Supplement: S2 Table — (DOCX) [file pone.0269822.s002.docx]

S2B According to the numerical calculation model, the three-zone spontaneous combustion in the goaf under different pressure differences between the upper and lower wells obtained from the simulation

| Different pressure between ground surface and working face/Pa | Distance between heat dissipation zone and working surface(m) | Distance between oxidation temperature rise zone and working surface(m) |
| --- | --- | --- |
| 800 | 38 | 102 |
| 600 | 26 | 96 |
| 400 | 19 | 78.5 |
| 200 | 10 | 28 |
| 0 | 4 | 9.5 |
| -200 | 15 | 33 |
