# Supplementary material for: Study on the air leakage characteristics of a goaf in a shallow coal seam and spontaneous combustion prevention and control strategies for residual coal
Source: PLoS One. 2022 Jun 24;17(6):e0269822. doi: 10.1371/journal.pone.0269822 (PMC9232134; doi:10.1371/journal.pone.0269822)
Supplement: S3 Table — (DOCX) [file pone.0269822.s003.docx]

S3C According to the field measurement results, the air leakage of the working face and the CO concentration in the upper corner during the pressurization process are obtained.

| Pressure difference between ground surface and working face(Pa) | Air leakage of working face(m^3^/s) | Carbon monoxide concentration(ppm) |
| --- | --- | --- |
| 862 | 4.5 | 59.07 |
| 537 | 2.74 | 82.44 |
| 470 | 2.39 | 71.83 |
| 341 | 1.93 | 62.13 |
| 217 | 1.25 | 72 |
| 112 | 0.74 | 18.15 |
| -85 | -0.54 | 27.8 |
| -178 | -1.14 | 25.92 |
